# *Anaplasma phagocytophilum* Community-Acquired Pneumonia: Case Report and Literature Review

**DOI:** 10.3390/microorganisms11061483

**Published:** 2023-06-02

**Authors:** Igor Dumic, Emily Person, Oladapo Igandan, Omobolanle Adetimehin, Charles W. Nordstrom, Christopher Williams, Fnu Shweta

**Affiliations:** 1 Mayo Clinic Alix School of Medicine, Rochester, MN 55905, USA; igandan.oladapo@mayo.edu (O.I.); adetimehin.omobolanle@mayo.edu (O.A.); nordstrom.cw@mayo.edu (C.W.N.); williams.christopher@mayo.edu (C.W.); shweta.fnu@mayo.edu (F.S.); 2 Department of Hospital Medicine, Mayo Clinic Health System, Eau Claire, WI 54703, USA; 3 Department of Emergency Medicine, Mayo Clinic, Rochester, MN 55905, USA; person.emily@mayo.edu; 4 Department of Pulmonary Medicine, Mayo Clinic Health System, Eau Claire, WI 54703, USA; 5 Department of Infectious Disease, Mayo Clinic Health System, Eau Claire, WI 54703, USA

**Keywords:** pneumonia, pneumonitis, *Anaplasma phagocytophilum*, Lyme disease, tick-borne diseases

## Abstract

*Anaplasma phagocytophilum* is an emerging, Gram-negative, and obligate intracellular pathogen that is infrequently implicated as a causative agent of community-acquired pneumonia. In this paper, we report about an immunocompetent patient from the community who presented with fever, cough, and shortness of breath. Chest X-ray and CT showed bilateral lung infiltrates. Extensive workup for other common and uncommon causes of pneumonia was positive for anaplasmosis. The patient recovered completely with doxycycline therapy. In our literature review, we find that in 80% of reported cases of anaplasmosis pneumonia, empiric treatment did not contain doxycycline, which in some cases led to acute respiratory distress syndrome. Clinicians in tick-borne disease endemic regions should be aware of this unusual presentation of anaplasmosis in order to be able to select appropriate antimicrobial regimens and initiate timely management.

## 1. Introduction

Human granulocytic anaplasmosis (HGA) refers to an acute infection caused by the obligate intracellular Gram-negative bacterium *Anaplasma phagocytophilum* [1,2,3]. *A. phagocytophilum* is most commonly transmitted through a tick vector [1]; however, transmission via blood transfusions [1,2,3] and other bodily fluids have been documented [1,2,3,4,5].

The clinical presentation of HGA may vary from an asymptomatic self-limited infection to a life-threatening infection with multiorgan involvement. Common manifestations include nonspecific symptoms such as fever, gastrointestinal complaints (most commonly diarrhea), headaches, myalgias, and arthralgias [1,2,3]. In addition to these nonspecific symptoms, HGA might also present with myocarditis, rhabdomyolysis, splenic rupture, trigeminal neuralgia, cerebral infarct, dysarthria, peripheral neuropathy, orchitis, Sweet syndrome or hemophagocytic lymphohistiocytosis (HLH), among others [1,2,3,4,5,6,7,8,9,10,11,12,13,14,15].

Respiratory complications and/or manifestations of HGA, however, are rare and seldom reported [15,16,17,18,19,20,21].

## 2. Case Description

During the summer of 2022, a 78-year-old woman from rural Wisconsin presented to urgent care for a three-week history of progressive fatigue, intermittent fever up to 39.5 °C, arthralgias, myalgias, nausea without vomiting, subjective dyspnea, and non-productive cough. She denied experiencing symptoms of headache, rhinorrhea, rash, chest pain, abdominal pain, dysuria, or diarrhea. Her past medical history was significant only for hypertension, hyperlipidemia, and gastroesophageal reflux disease. She denied having any allergies and had no history of tobacco, alcohol, or illicit drug use. She did report owning a pet dog and recalled removing several ticks from both the dog and herself approximately one week prior to symptom onset. The patient reported that she spends a significant amount of time during summer months outdoors hiking, riding a bicycle, and gardening. She denied any travel outside of Wisconsin at least a year prior to her hospitalization.

On presentation, she was found to be a well-developed elderly woman without distress. Her sensorium was clear, and she was able to answer questions appropriately. Vitals demonstrated tachycardia with a heart rate of 130 beats per minute, but she was otherwise afebrile, normotensive, with normal respiration, and saturating appropriately on room air. On physical examination, the only finding was mild epigastric tenderness on palpation, but without guarding or rebound, and bowel sounds were normoactive. The remainder of her exam was unremarkable with moist oral mucosa, anicteric sclerae, supple neck, normal heart tones, clear lungs, and no rash was detected.

Laboratory evaluation was notable for profound thrombocytopenia of 22,000 (normal range: 157,000–371,000), marked elevation in C-reactive protein (CRP) of 250.4 mg/L (normal range: <5 mg/L), and elevated liver function tests with alanine aminotransferase (ALT) of 129 U/L (normal range: 7–45), aspartate aminotransferase (AST) of 128 U/L (normal range: 8–43), alkaline phosphatase of 127 U/L (normal range 35–104), and total bilirubin of 2.2 mg/L (normal range: <1.2). Normal values were demonstrated for white blood cell count, hemoglobin, electrolytes, kidney function, and lipase. Testing for severe acute respiratory syndrome coronavirus (SARS-CoV-2) using polymerase chain reaction (PCR) of nasal swabs and serologies for hepatitis A, B, and C were all negative. Trends of basic anaplasmosis-specific labs are illustrated in Table 1.

**Figure 1:**
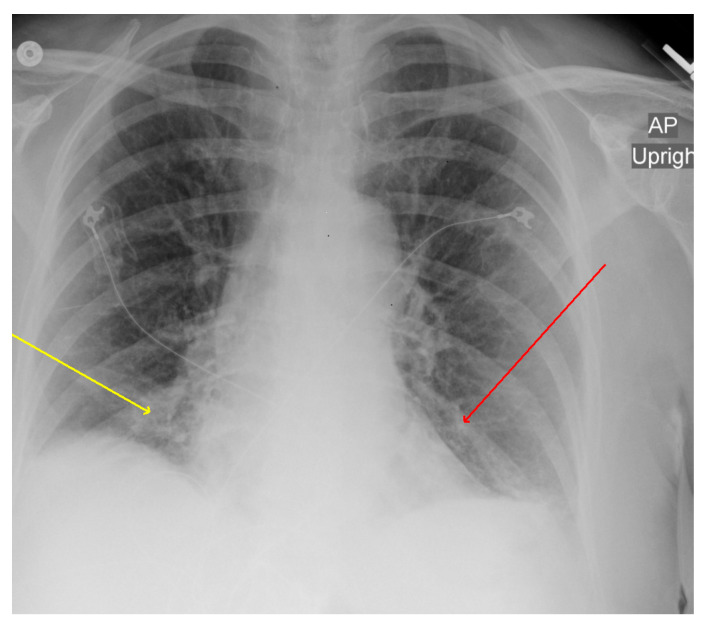
Chest X-ray (CXR): AP chest X-ray showing bibasilar infiltrates; right (yellow arrow) greater than left (red arrow).

**Figure 2:**
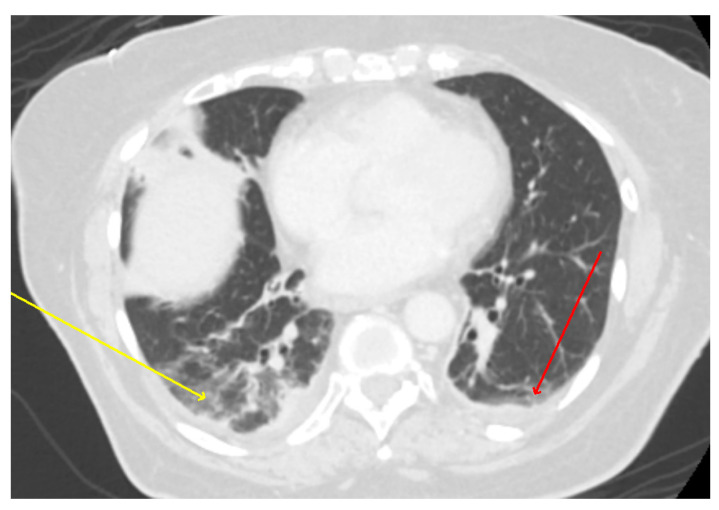
CT scan: CT abdomen showing lower lung fields with bibasilar ground-glass opacities; right (yellow arrow) greater than left (red arrow) with small bilateral pleural effusions versus atelectasis.

An ultrasound of the gallbladder and biliary ducts did not show any abnormality. A chest X-ray (CXR) demonstrated patchy bibasilar opacities, which were concerning for pneumonia (Figure 1). Computerized tomography (CT) scan of the abdomen and pelvis with contrast did not show any acute intraabdominal abnormalities but demonstrated trace bilateral pleural effusions with bibasilar ground glass opacities which were more severe within the right lung (Figure 2).

The patient was started on empiric treatment with IV ceftriaxone and doxycycline for presumptive community-acquired pneumonia (CAP) due to her clinical presentation in conjunction with her radiographic and laboratory findings. Doxycycline was chosen over IV azithromycin to provide empiric coverage for tick-borne illnesses given her thrombocytopenia, transaminitis, and epidemiological information (tick exposure while living in an endemic area).

A tick-borne illness panel was performed at Mayo Medical Laboratories (we use a modified 2-tier serologic testing for tick-borne infections. Initial test detects *Anaplasma phagocytophilum*, *Ehrlichia chaffeensis*, *Babesia microti* (species-specific immunofluorescence assay (IFA) IgG for each, DiaSorin Molecular Cypress, CA, USA), and Lyme disease (enzyme-linked immunosorbent assay (ELISA, Helsinki, Finland); Borrelia VlsE1/pepC10 IgG/IgM Test System, Zeus Scientific, Inc., Branchburg, NJ, USA). Because this test does not differentiate between IgM and IgG antibodies, any positive/equivocal result for Lyme antibody on this multiplex panel is reflexed to confirmation on separate ELISA (ZEUS ELISA *Borrelia burgdorferi* IgM and IgG Test System) for IgG and IgM obtained on admission returned positive for antibodies against *A. phagocytophilum* (both positive for IgM and IgG (>1:2048) and antibodies against *Borrelia burgdorferi* were IgM positive and IgG negative. PCR for *Anaplasma phoagocytophilum* returned positive as well. A peripheral blood smear obtained on day 3 of admission showed an absolute lymphocytosis with reactive lymphocyte morphology with rare neutrophil inclusions consistent with anaplasmosis. Moderate thrombocytopenia was confirmed on the smear as well. Blood culture (collected into 2 BACTEC Plus Aerobic/F bottles and 1 BACTEC Lytic/10 Anaerobic/F bottle and then incubated into the automated BACTEC FX instrument for 5 days. Antimicrobial susceptibility testing is performed on the Vitek 2 system) and sputum cultures remained without growth. Broad testing for common viral and bacterial pathogens via nasopharyngeal PCR testing returned negative, including Influenza A, Influenza B, respiratory syncytial virus (RSV), *Mycoplasma pneumoniae*, and *Chlamydia pneumoniae*. Additionally, urine *Legionella pneumophila* and *Streptococcus pneumoniae* antigens were also negative. The full list of laboratory tests performed to exclude other causes of CAP is listed in Table 2.

Within 24 h of initiating antibiotic therapy, the patient markedly improved. By hospital day 5, the patient’s liver enzymes and thrombocytopenia have improved, and inflammatory markers have significantly trended down. Clinically, her shortness of breath had resolved, and her cough had markedly improved. She was discharged home to complete 21 days of doxycycline.

## 3. Discussion

The incidence of tick-borne infections, including anaplasmosis, has increased significantly over the last two decades [22]. Climate change leading to warmer winters and increased precipitation has contributed to an increase in tick survival and availability to human hosts. This trend in climate has led to a prediction that tick-borne infections will increase by at least 20% in the coming decade [23]. In the United States, the main tick vector for human disease is *Ixodes scapularis* within the Northeast and the Midwest and *Ixodes pacificus* in the Pacific Northwest of the country. These ticks can transmit at least five other bacteria in addition to *A. phagocytophilum* (*Borrelia mayonii*, *Borrelia miyamotoi*, *Ehrlichia muris eauclairensis, Ehrlichia chaffeensis* and *Borrelia burgdorferi)*, one parasite (*Babesia microti*), and the Powassan virus [24]. In Europe, *Ixodes ricinus* was also demonstrated to be a suitable vector for *Bartonella* spp. transmission [25]. Ticks can frequently transmit more than one pathogen with co-infection leading to more serious clinical presentations and complicated diagnoses [26]. In the United States there are geographical differences in the rate of tick co-infection with these pathogens leading to a higher incidence within the Northeast when compared to the Midwest [26].

Our patient was diagnosed with anaplasmosis based on the results of laboratory investigation. While early localized Lyme disease remained a possibility, it was felt to be the less likely cause of pneumonia based on the evidence from the literature, where lung involvement was more commonly reported with anaplasmosis. 

We performed the search of PubMed/MEDLINE using the keywords pneumonia and pneumonitis and Lyme disease and anaplasmosis. We identified nine more reports of pulmonary involvement in the context of anaplasmosis but only one case report of pulmonary involvement in a patient with Lyme disease [15,16,17,18,19,20,21,27]. As illustrated in Table 3, anaplasmosis can lead to various respiratory symptoms and radiographic findings. The most common respiratory symptoms appear to be cough and shortness of breath. While CXR and CT may demonstrate interstitial infiltrates and pleural effusions which are typically bilateral, in rare cases acute respiratory distress syndrome (ARDS) has been reported [15,20]. The sole case report we identified through our literature search for pulmonary involvement with Lyme disease was a patient who had fatal pulmonary involvement from ARDS [27]. The remainder of other pulmonary manifestations described in patients with Lyme disease were secondary to the cardiac complications which are quite common in the early disseminated stage of *B. burgdorferi* infection (for example, pulmonary edema from heart failure due to Lyme carditis) [28,29]. Phrenic nerve paralysis in neuroborreliosis leading to respiratory failure has been described as well; however, respiratory failure in such cases is indirect and not caused by lung parenchymal damage [29]. As such, in our case, we believe that pneumonia was caused by anaplasmosis rather than Lyme disease.

Patients such as ours who present from the community with lung infiltrates, cough, abnormal liver enzymes, nausea, thrombocytopenia, and elevated inflammatory markers present a unique challenge from a diagnostic perspective. Occupational risk, place of residence, pet exposure, and travel history are all important to assess in such scenarios. Common as well as unusual diseases, both infectious and noninfectious, must be considered. Exclusion of the most common bacterial and viral causes of community-acquired pneumonia while providing empiric antimicrobial treatment based on the current guidelines remains the first step [30]. While *Legionella pneumophila* can present with transaminitis and gastrointestinal symptoms, thrombocytopenia is not a prominent feature. In tick-endemic areas, the presence of pneumonia in combination with thrombocytopenia might be due to ehrlichiosis, anaplasmosis, babesiosis, tularemia, or Rocky Mountain spotted fever (RMSF). Pneumonia related to babesiosis has been described but remains rare [31]. Babesiosis usually presents with some degree of hemolytic anemia which was absent in our patient, making that diagnosis unlikely. Tularemia can present with pneumonia in about 40% of adult cases, but thrombocytopenia is not a common finding [32]. Patients with ehrlichiosis are usually sicker than patients with anaplasmosis, but more commonly present with a rash and less commonly thrombocytopenia [33]. *Ehrlichia chaffeensis* is transmitted by the Lone Star tick (*Amblyomma americanum*) with a geographical predominance in the southeastern and south-central USA rather than the northern Midwest. RMSF can present identically to our patient, but a rash is present in almost 80% of cases [34] and was absent in our patient. Except for babesiosis, treatment with doxycycline is effective against all the above-mentioned pathogens.

Doxycycline remains the first line treatment for anaplasmosis, and thus far no antimicrobial resistance of *A. phagocytophilum* to doxycycline has been reported [33,35]. It is important to highlight (Table 3) that in 80% of cases of anaplasmosis who presented with respiratory symptoms and radiographic findings of pneumonia empiric treatment did not include doxycycline. In some cases [17,19], levofloxacin proved to be efficacious; however, while it is reassuring that levofloxacin does appear to have activity against *A. phagocytophilum* in vitro, it has not been evaluated for the treatment of anaplasmosis in any of the clinical trials to date.

In some cases, where the empiric antimicrobial regimen did not include doxycycline patients quickly progressed to respiratory failure [15]; however, all such cases of respiratory failure resolved once appropriate therapy with doxycycline was initiated. It is worth highlighting that ARDS can sometimes complicate anaplasmosis. One case report [20] described severe ARDS from anaplasmosis that was complicated with clinical features resembling hemophagocytic lymphohistiocytosis (HLH). In this case, recovery was attained by the use of intravenous steroids.

Treatment for anaplasmosis, including cases of pneumonia, has never been studied in clinical trials. From Table 2 we can see that the majority of the cases were treated for 10 days. In cases of co-infection and the presence of Lyme disease, treatment with doxycycline should be extended to 21 days. In cases where there is neurological or cardiac involvement, ceftriaxone is necessary in the acute phase [35].

## 4. Conclusions

In summary, we present a case of *Anaplasma phagocytophilum* in a patient with community-acquired pneumonia. Doxycycline could be considered as a preferred atypical antibacterial coverage in patients presenting with CAP in areas endemic for tick-borne illnesses with epidemiologic risk factors consistent with significant exposure to tick bites. At a minimum, clinicians should maintain a high clinical suspicion for tick-borne illness and initiate prompt evaluation when appropriate to ensure treatment when indicated. By reporting this case, we want to raise awareness among clinicians in endemic areas about this unusual presentation of the disease.

## Figures and Tables

**Table 1:** Illustrates the trend of common laboratory derangement seen in patients with anaplasmosis. Abnormal values are highlighted in red.

	Admission	Day 2	Discharge
Hemoglobin	12.4	11.9	11.6
Platelet Count	22	38	69
White Blood Cell Count	4.3	9.2	7.9
Neutrophils	2.15	1.07	0.91
Lymphocytes	1.94	6.72	6.21
Bilirubin, Total	2.2	0.3	0.8
Alanine Aminotransferase	129	107	109
Aspartate Aminotransferase	128	114	100
C-reactive Protein	250.4	75.9	41.2

**Table 2:** This table illustrates extensive workup that was carried out for diagnosis of community-acquired pneumonia in our patient.

Test	Blood Specimen	Urine Specimen	Sputum Specimen	Respiratory Nasopharyngeal Swab
Cultures	Negative	Negative	Negative	
*Legionella pneumophila* antigen	-	Negative	-	-
*Streptococcus pneumonia* antigen	-	Negative	-	-
Peripheral smear	Intra neutrophilic inclusion	-	-	-
*Mycoplasma pneumonia* antibodies	IgM and IgG negative	-	-	-
*Chlamydia pneumoniae* antibodies	IgM negative	-	-	-
*Chlamydia trachomatis* antibodies	IgM and IgG negative	-	-	-
*Chlamydia psittaci* antibodies	IgM and IgG negative	-	-	-
*Adenovirus*	-	-	-	Negative
*Coronavirus* 229E	-	-	-	Negative
*Coronavirus* HKU1	-	-	-	Negative
*Coronavirus* NL63	-	-	-	Negative
*Coronavirus* OC43	-	-	-	Negative
*SARS Coronavirus*-2	-	-	-	Negative
*Human Metapneumovirus*	-	-	-	Negative
*Human Rhinovirus/Enterovirus*	-	-	-	Negative
*Influenza A Virus*	-	-	-	Negative
*Influenza B Virus*	-	-	-	Negative
*Parainfluenza Virus* 1	-	-	-	Negative
*Parainfluenza Virus* 2	-	-	-	Negative
*Parainfluenza Virus* 3	-	-	-	Negative
*Parainfluenza Virus* 4	-	-	-	Negative
*Respiratory Syncytial Virus*	-	-	-	Negative
*Bordetella parapertussis*	-	-	-	Negative
*Bordetella pertussis*	-	-	-	Negative
*Chlamydia pneumoniae*	-	-	-	Negative
*Mycoplasma pneumoniae*	-	-	-	Negative
*Borrelia burgdorferi*	IgM positive	-	-	-
*Anaplasma phagocytophilum*	Positive IgG, IgM, and PCR	-	-	-
*Borrelia miyamotoi*, PCR, B	Negative	-	-	-
*Ehrlichia chaffeensis*	Negative	-	-	-
*Ehrlichia ewingii/canis*	Negative	-	-	-
*Ehrlichia muris eauclairensis*	Negative	-	-	-
*Babesia divergens*/MO-1	Negative	-	-	-
*Babesia duncani*	Negative	-	-	-

**Table 3:** This table summarizes previously published cases of anaplasmosis presenting with pneumonia, including patients’ symptoms, radiological findings, empiric antimicrobial regimen, and the outcome.

Case Reference	Age	Sex	Country Year	Respiratory Symptoms	Imaging	Treatment (Duration in Days)	Appropriate Empiric Treatment	Outcome
1 [18]	76	M	USA 2001	Nonproductive Cough	Bilateral pleural effusions and patchy GGO	DOX (3) LEV(8)	Yes	Improved
2 [17]	47	M	France 2003	Cough	Bilateral interstitial infiltrates	Spiramycin Cefpodoxime (15)	No	Improved
3 [21]	77	M	USA 2011	Dyspnea Hypoxia	Left lung atelectasis, left pleural effusion	DOX (10)	No	Improved
4 [20]	62	M	USA 2015	Dyspnea	Bilateral patchy GGO, ARDS	DOX IV steroids	Yes	Improved
5 [15]	70	F	USA 2020	Cough Dyspnea	Bilateral GGO	DOX (10)	No	Improved
6 [15]	63	F	USA 2020	Cough Dyspnea	Right upper and middle lobe GGO	DOX (10)	No	Improved
7 [15]	64	M	USA 2020	Dyspnea Chest Pain	Peripheral GGO bilaterally	DOX (10)	No	Improved
8 [15]	78	M	USA 2020	Productive cough Dyspnea	Bilateral pleural effusion	Dox (21)	No	Improved
9 [16]	57	M	Turkey 2022	Cough Tachypnea	Bilateral opacities	DOX (18)	No	Improved
10 [19]	56	F	USA 2022	Dry Cough	Bilateral GGO and pleural effusions	DOX (10)	No	Improved
Patient from this report	78	F	USA 2020	Cough Hypoxia	Bilateral infiltrates and bilateral pleural effusion	DOX (21)	Yes	Improved

ARDS—acute respiratory distress syndrome; GGO—ground-glass opacity. M—male; F—female; DOX—doxycycline; LEV—levofloxacin.

## Data Availability

Available from corresponding author upon request.
